# Evaluating the Effects of Nanocurcumin Supplementation in Type 2 Diabetes Mellitus: A Systematic Review and Meta‐Analysis of Randomized Controlled Trials

**DOI:** 10.1002/edm2.70242

**Published:** 2026-05-22

**Authors:** Sahar Ghoflchi, Mohammad Jalili‐Nik, Seyed Isaac Hashemy, Sercan Karav, Hossein Hosseini, Amirhossein Sahebkar

**Affiliations:** ^1^ Department of Clinical Biochemistry, Faculty of Medicine Mashhad University of Medical Sciences Mashhad Iran; ^2^ Department of Molecular Biology and Genetics Canakkale Onsekiz Mart University Canakkale Turkey; ^3^ Biotechnology Research Center Pharmaceutical Technology Institute, Mashhad University of Medical Sciences Mashhad Iran; ^4^ Centre for Research Impact & Outcome, Chitkara College of Pharmacy, Chitkara University Rajpura Punjab India; ^5^ Applied Biomedical Research Center Basic Sciences Research Institute, Mashhad University of Medical Sciences Mashhad Iran

**Keywords:** glycemic control, inflammation, lipid profile, meta‐analysis, nanocurcumin, oxidative stress, type 2 diabetes

## Abstract

**Background:**

Nanocurcumin has been developed to overcome the poor bioavailability of conventional curcumin and has been proposed as a potential adjunctive therapy for type 2 diabetes mellitus (T2DM). However, evidence regarding its clinical efficacy remains inconsistent. This systematic review and meta‐analysis aimed to evaluate the effects of nanocurcumin supplementation on glycemic control, lipid profile, inflammatory markers and oxidative stress in patients with T2DM.

**Methods:**

A systematic search of PubMed, Scopus and the Cochrane Library was conducted from inception to October 2025 to identify randomized controlled trials (RCTs) assessing nanocurcumin supplementation in adults with T2DM. Eligible studies compared nanocurcumin with placebo or no intervention and reported outcomes related to glycemic indices, lipid parameters, inflammatory markers or oxidative stress. Pooled effect sizes were calculated using a random‐effects model and expressed as standardized mean differences (SMDs) with 95% confidence intervals (CIs). Between‐study heterogeneity was assessed using the *I* ^2^ statistic.

**Results:**

Seven RCTs involving 453 participants were included. Nanocurcumin supplementation showed no significant effects on fasting blood glucose (SMD: −0.44; 95% CI: −2.16 to 1.28), HbA1c (SMD: 0.19; 95% CI: −1.26 to 1.65), or lipid profile parameters, including total cholesterol, LDL‐C, HDL‐C and triglycerides (all *p* > 0.05). No statistically significant reduction in C‐reactive protein was observed (SMD: −0.78; 95% CI: −1.58 to 0.05). In contrast, nanocurcumin significantly increased total antioxidant capacity (SMD: 1.60; 95% CI: 0.93 to 2.31) and reduced malondialdehyde levels (SMD: −1.93; 95% CI: −3.19 to −0.66), indicating a robust antioxidative effect.

**Conclusions:**

Current evidence suggests that short‐term nanocurcumin supplementation does not significantly improve glycemic control, lipid profile or systemic inflammation in patients with T2DM. However, preliminary evidence suggests that nanocurcumin may improve oxidative stress markers, reflected by improved total antioxidant capacity and reduced oxidative stress. These findings indicate that the potential clinical utility of nanocurcumin in T2DM may be primarily related to its antioxidative properties rather than direct metabolic modulation. Larger, long‐term RCTs using standardized nanocurcumin formulations are warranted to determine its impact on diabetes‐related complications and clinical outcomes. Although the primary pooled analysis showed no significant effects on lipid profile and CRP, sensitivity analysis revealed that exclusion of the study by Mansour et al. (2025), which had a longer duration and older participants, resulted in statistically significant reductions in total cholesterol and CRP. Given the substantial heterogeneity and limited number of studies, subgroup analyses were not feasible, and findings should be interpreted with caution.

## Graphical abstract

This meta‐analysis of trials was conducted to assess the effect of nanocurcumin supplementation in type 2 diabetes mellitus.

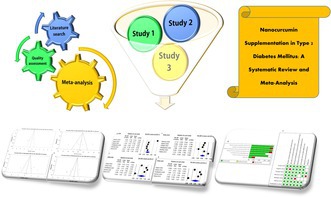

## 1 Introduction

Type 2 diabetes mellitus (T2DM) is a progressive and multifactorial metabolic disorder characterized by chronic hyperglycemia, insulin resistance and β‐cell dysfunction, and it accounts for nearly 90% of all diabetes cases worldwide [1, 2]. The global prevalence of T2DM has risen dramatically over the past decades, increasing from 6.4% in 2010 to 10.5% in 2021, with projections estimating a further rise to 12.2% by 2045 [1]. This escalation is strongly associated with ageing populations, obesity, sedentary lifestyles, and improved survival of diabetic patients due to advances in early detection and therapeutic management [2]. The pathophysiology of T2DM is complex, encompassing the ‘ominous octet’, which includes impaired insulin secretion, insulin resistance, altered incretin effect, hepatic glucose overproduction, adipose tissue dysfunction and neural dysregulation [3, 4, 5]. Moreover, oxidative stress and chronic low‐grade inflammation have emerged as pivotal contributors to β‐cell dysfunction and insulin resistance [6]. These mechanisms not only contribute to poor glycemic control but also accelerate the development of microvascular and macrovascular complications such as diabetic neuropathy, nephropathy, retinopathy and cardiovascular diseases [7, 8].

Curcumin, a polyphenolic compound derived from the rhizome of *Curcuma longa* (turmeric), has attracted considerable scientific interest over the past recent decades owing to its anti‐inflammatory, antioxidant, and hypoglycemic properties [9, 10, 11, 12, 13]. Experimental and clinical studies have demonstrated that curcumin reduces oxidative stress, improves lipid profiles, inhibits platelet aggregation and modulates insulin signalling, thereby mitigating several metabolic and vascular complications of diabetes [14, 15]. However, the therapeutic potential of conventional curcumin has been proposed to be hampered by its low solubility, rapid metabolism, and limited systemic bioavailability [16].

Nanocurcumin formulations, designed through nanotechnology, have been developed to overcome these pharmacokinetic limitations. By reducing curcumin to nanoscale particles, these formulations significantly enhance solubility, absorption and plasma retention, resulting in improved bioavailability and therapeutic efficacy [17, 18, 19, 20]. Multiple studies have reported that nanocurcumin exerts significant effects on glycemic control, lipid regulation, oxidative stress reduction and inflammation [16, 18, 21]. Thus, nanocurcumin represents a novel adjunctive strategy with the potential to improve both metabolic regulation and long‐term clinical outcomes in patients with T2DM [17, 21]. The objective of the present systematic review and meta‐analysis was to comprehensively evaluate the efficacy of nanocurcumin supplementation on lipid profile, glycemic biomarkers and oxidative stress markers in T2DM patients.

## 2 Methods

### 2.1 Search Strategy

A comprehensive literature search was conducted from database inception to October 2025 across Medline/PubMed, Scopus and the Cochrane Library to identify relevant randomized controlled trials. Based on the PICO framework, the population (P) was adults diagnosed with T2DM, the intervention (I) was nanocurcumin supplementation at any dosage, the comparison (C) was placebo or no intervention, and the outcomes (O) were glycemic biomarkers including fasting blood sugar (FBS), haemoglobin A1C (HbA1C), serum lipid parameters including total cholesterol, LDL‐C, HDL‐C and triglycerides, as well as total antioxidant capacity (TAC), malondialdehyde (MDA) and C‐reactive protein (CRP). Our search was carried out using Medical Subject Headings (MeSH) terms, which include (nanocurcumin OR curcumin nanoparticle) AND (Type 2 diabetes mellitus OR T2DM OR diabetes) AND (lipid profile OR cholesterol OR triglyceride OR HDL* OR LDL* OR fasting blood sugar OR FBS OR haemoglobin A1C OR HbA1C OR total antioxidant capacity OR TAC, OR malondialdehyd OR MDA, OR C‐reactive protein OR CRP OR hsCRP), AND (randomized controlled trial OR randomized controlled trial OR RCT). Search strategies were adapted for each database. Additionally, to ensure completeness, we searched the reference lists of the included studies and relevant reviews.

### 2.2 Screening and Data Extraction

Studies were included if they met all of the following criteria: (a) Adult participants diagnosed with type 2 diabetes mellitus (T2DM) based on accepted clinical or laboratory criteria; (b) randomized controlled trial (RCT) design, with parallel or crossover structure; (c) nanocurcumin used as the primary intervention, with the term ‘nanocurcumin’ explicitly mentioned in the title or abstract of the original article; (d) presence of a placebo or no intervention control group; (e) reporting pre‐ and post‐intervention data for at least one of the following outcomes: glycemic biomarkers (FBS and HbA1c), lipid profile (TC, LDL‐C, HDL‐C and TG), oxidative stress markers (TAC and MDA), inflammatory markers (CRP or hs‐CRP) and (f) full‐text availability in English. This author‐defined terminology was used as an operational criterion because most clinical trials did not provide consistent physicochemical characterization (e.g., particle size, PDI and encapsulation method), preventing the use of laboratory‐based definitions of nanocurcumin.

Studies were excluded if they met any of the following conditions: (a) lack of post‐intervention data, or reporting baseline values only; (b) use of active comparators (e.g., other supplements and medications) instead of placebo or no‐treatment control; (c) non‐English publications or lack of accessible full text; (d) non‐clinical studies, including: in vitro experiments, animal studies, pilot studies without control groups, case reports, conference abstracts, letters, book chapters, review articles, protocols without results; (e) studies using curcumin formulations that were not designated as ‘nanocurcumin’ by the authors in the title or abstract; (f) studies with major methodological flaws, including unclear randomization, absence of control group or incomplete outcome reporting.

### 2.3 Data Extraction and Quality Evaluation

Data extraction was conducted independently by two reviewers employing a standardized data collection template. Any inconsistencies were addressed through discussion with a third reviewer. For quality evaluation, the following study characteristics were retrieved: name of the first author, year of publication, study design, sample size of both intervention and control groups, as well as the mean values with their corresponding standard deviation (SD) of the reported outcome measures. Due to incomplete reporting in several of the included trials, detailed physicochemical properties of nanocurcumin formulations (particle size, encapsulation matrix, curcuminoid purity and bioavailability data) could not be fully extracted. Although these factors are known to influence pharmacokinetics, inconsistent reporting prevented meaningful comparative analysis between formulation types.

### 2.4 Risk of Bias Assessment and Sensitivity Analyses

The methodological quality of the included RCTs was evaluated using the Cochrane Risk of Bias tool, which includes seven domains included random sequence generation, allocation concealment, blinding of participants and personnel, blinding of outcome assessment, completeness of data analysis, selective outcome reporting and other potential sources of bias. Based on the assessment results, studies were categorized into three levels of methodological quality: low risk of bias, defined as having all domains rated as ‘low risk’; moderate risk of bias, characterized by at least one domain rated as ‘unclear risk’; and high risk of bias, indicated by the presence of at least one domain rated as ‘high risk’ [22].

Quality assessments were performed using Review Manager (RevMan), the Cochrane Collaboration's official software for systematic reviews. In addition, leave‐one‐out sensitivity analyses were conducted by sequentially excluding individual studies to examine the robustness of the overall findings and to ensure that no single study unduly influenced the results. Funnel plots were generated for outcomes with at least five studies to visually assess publication bias (Figures S1 and S1), acknowledging the limited statistical power [22]. Therefore, the possibility of publication bias cannot be excluded.

### 2.5 Statistical Analysis

All statistical analyses were performed using Comprehensive Meta‐Analysis software (Biostat, NJ) and OpenMeta [Analyst], version 24.1. For each outcome, effect sizes were expressed as standardized mean differences (SMDs) with corresponding 95% confidence intervals (CIs), thereby enabling comparisons across studies employing different measurement scales. To address potential variability among studies, a random‐effects model was employed. Between‐study heterogeneity was evaluated using both Cochran's *Q*‐test (with a threshold of *p* < 0.1 considered significant) and the *I* ^2^ statistic, with values of 25%, 50% and 75% representing low, moderate and high heterogeneity, respectively (Table 2).

## 3 Result

### 3.1 Study Selection

**FIGURE 1:**
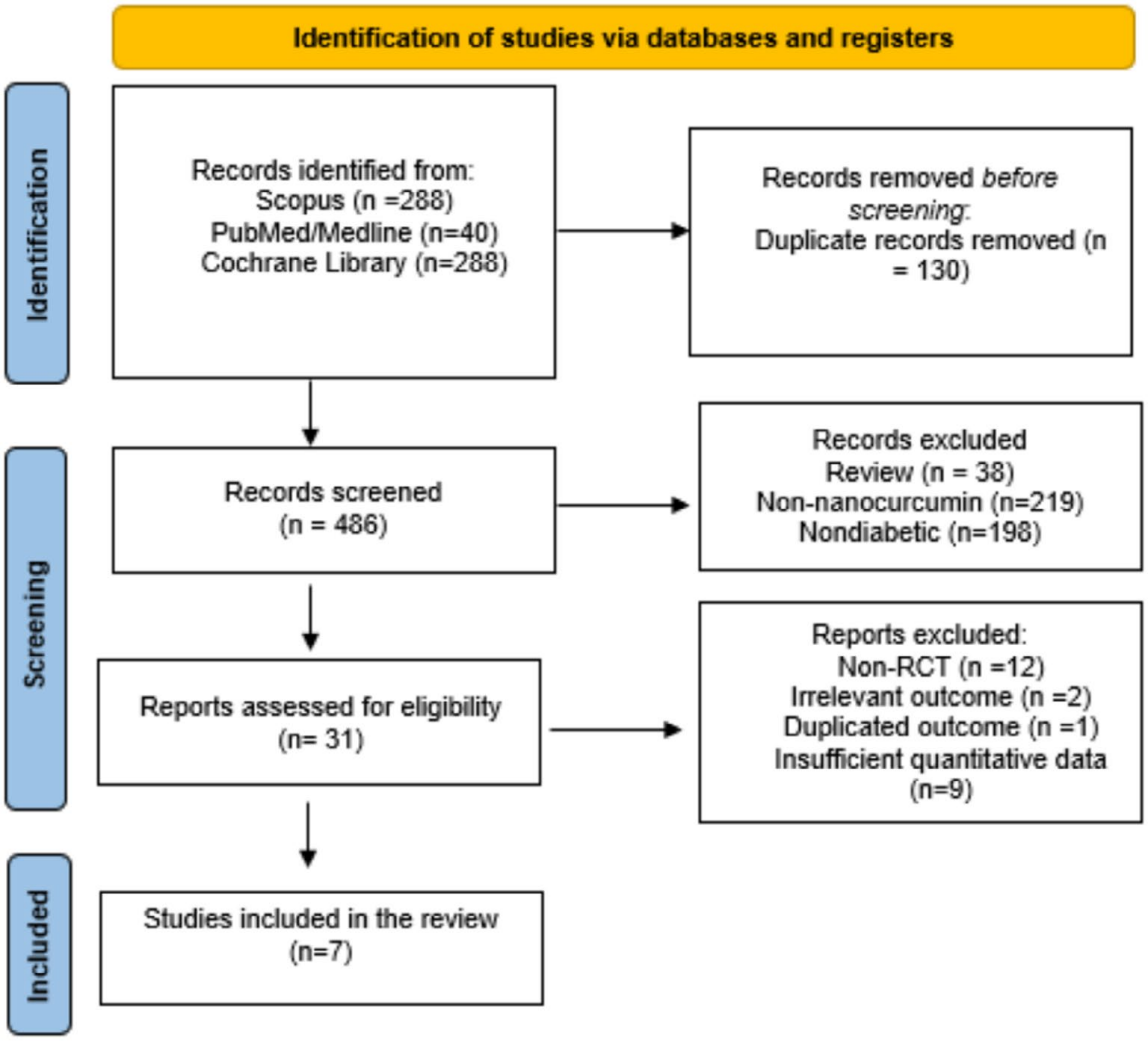
PRISMA flow chart to illustrate the article search and selection process.

A total of 616 records relevant to the research topic were initially retrieved from all searched databases. After removing duplicates and discarding unrelated records, 486 articles remained for preliminary screening. During the title and abstract screening phase, 455 records were excluded due to Review type, nanocurcumin administration or nondiabetic disease. Subsequently, 31 full‐text articles were assessed for eligibility assessment, during which 24 studies were excluded for various reasons, including inappropriate study design, irrelevant outcomes, duplicated outcomes and insufficient quantitative data. Ultimately, 7 studies satisfied all predefined inclusion criteria and were incorporated into the final systematic review and meta‐analysis [21, 23, 24, 25, 26, 27, 28]. The entire study selection process is visually summarized in the PRISMA flow diagram (Figure 1), while the demographic and methodological characteristics of the included studies are presented in Table 1. Detailed characteristics of the nanocurcumin formulations used in the included trials are presented in Table 3.

**TABLE 1:** Characteristics of studies included in the systematic review and meta‐analysis.

Author (Ref.)	Year	Design	Age. intervention	Age. control	Dose (mg/day)	N. intervention	N. control	Follow‐up (weeks)
Mansour [27]	2025	RCT	62.32	62.67	80	41	45	16
Asghari [23]	2024	RCT	54.56	57.48	80	25	25	12
Dastani [28]	2023	RCT	60	60.53	80	32	32	12
Shafabakhsh [24]	2020	RCT	58.3	56.2	80	26	27	12
Mokhtari [25]	2020	RCT	55	55.8	80	25	25	12
Asadi [26]	2019	RCT	53.6	54.6	80	40	40	8
Rahimi [21]	2015	RCT	56.34	60.95	80	35	35	12

### 3.2 Quality Assessment of Included Studies

**FIGURE 2:**
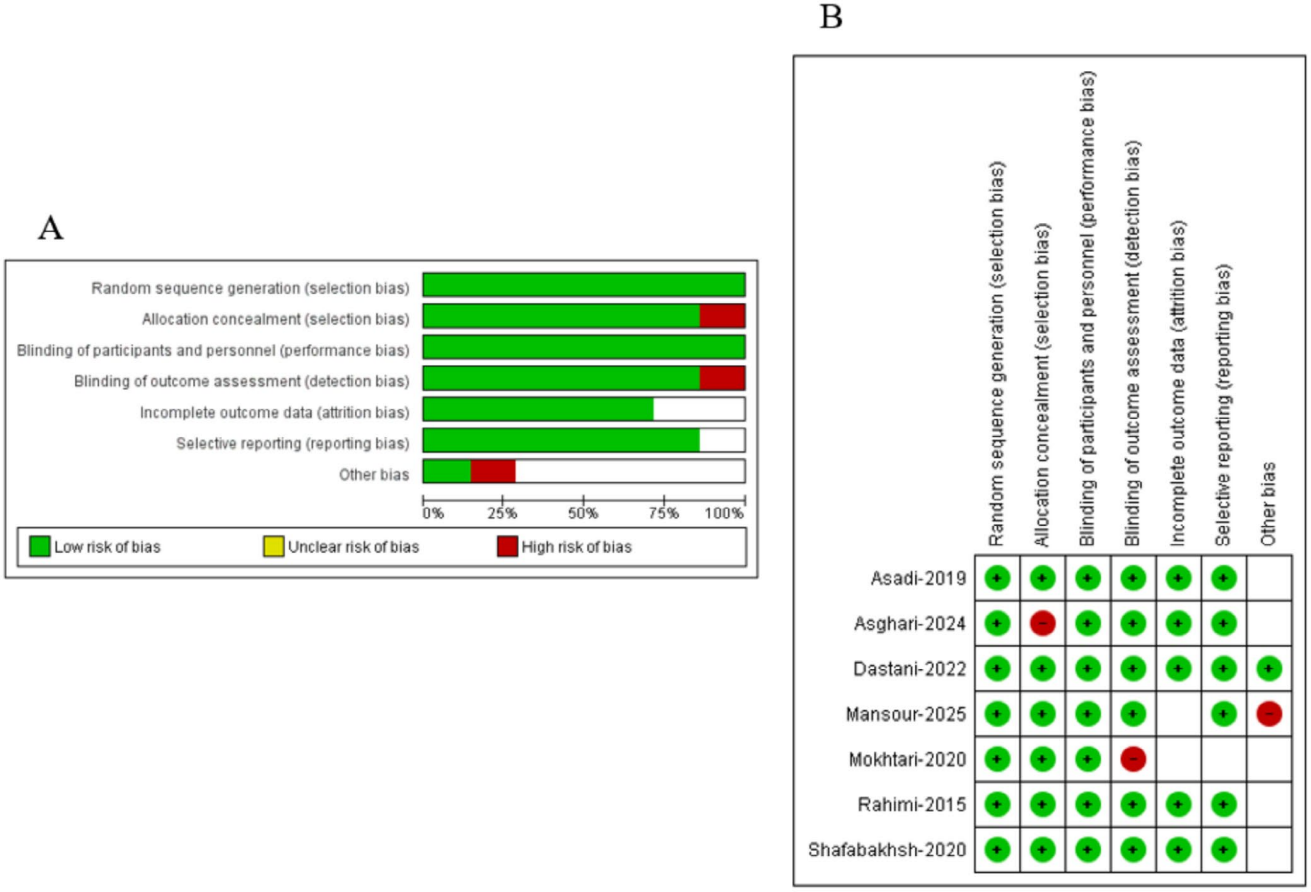
Summary and detailed evaluation of the risk of bias in the included studies. (A) presents the overall summary of bias risk across all studies and (B) presents the domain‐specific assessment for each study. A green dot indicates a low risk of bias, a wight dot denotes an unclear risk of bias, and a red dot indicates a high risk of bias.

The methodological quality of the included RCTs was critically appraised to ensure the internal validity and reliability of the meta‐analysis findings. Risk of bias was assessed using the Cochrane Risk of Bias tool. Based on this assessment, all included studies were rated as having a low overall risk of bias. The results of the quality assessments are graphically depicted in Figure 2. Sensitivity analyses for some parameters showed that omitting individual studies did not significantly alter the pooled estimates (Figures 6, 7, 8).

### 3.3 Effect of Nanocurcumin on Primary Outcomes

#### 3.3.1 Total Cholesterol

**FIGURE 3:**
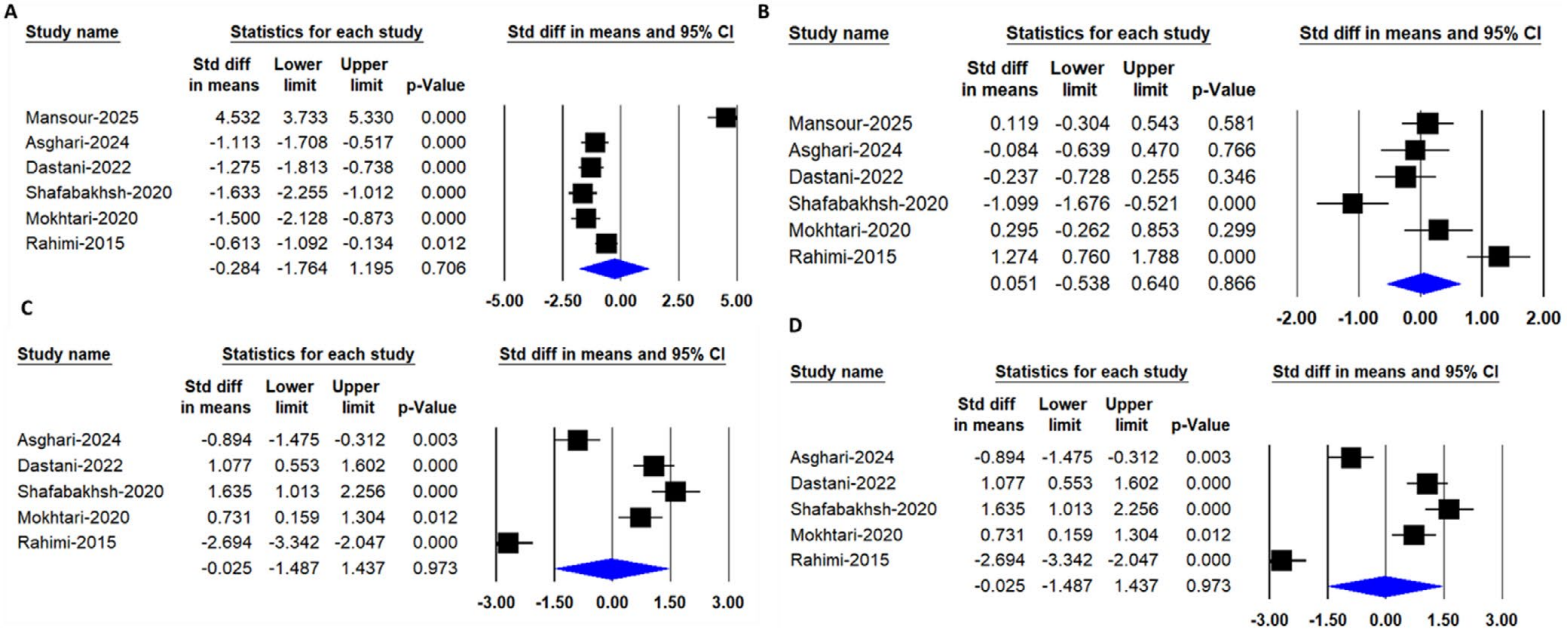
Forest plots demonstrating the pooled effects of nanocurcumin supplementation on lipid profile parameters, including (A) total cholesterol, (B) triglycerides, (C) low‐density lipoprotein cholesterol (LDL‐C) and (D) high‐density lipoprotein cholesterol (HDL‐C) levels.

This random‐effects meta‐analysis, which included 6 RCTs encompassing 373 participants (184 in the intervention group and 189 in the control group), demonstrated no significant reduction in total cholesterol levels following nanocurcumin supplementation [SMD: −0.28, 95% CI: (−1.76, 1.19), *p* = 0.70] (Figure 3).

#### 3.3.2 Triglyceride

This random‐effects meta‐analysis included 373 people from 6 clinical trials (184 cases and 189 controls). Results showed that consuming nanocurcumin did not significantly reduce triglyceride levels [SMD: 0.05, 95% CI: (−0.53, 0.64), *p* = 0.86] (Figure 3).

#### 3.3.3 HDL‐Cholesterol

A random‐effects meta‐analysis that included 6 trials with a total of 373 participants (184 cases and 189 controls) found no significant difference in HDL‐C levels after consuming nanocurcumin [SMD: −0.02, 95% CI: (−1.48, 1.43), *p* = 0.97] (Figure 3).

#### 3.3.4 LDL‐Cholesterol

Nanocurcumin administration did not significantly reduce LDL‐C levels, as shown by a random‐effects meta‐analysis of 6 trials involving 373 participants (184 in the intervention group and 189 in the control group) [SMD: 0.03, 95% CI: (−1.39, 1.45), *p* = 0.96] (Figure 3).

#### 3.3.5 Fasting Blood Glucose

**FIGURE 4:**
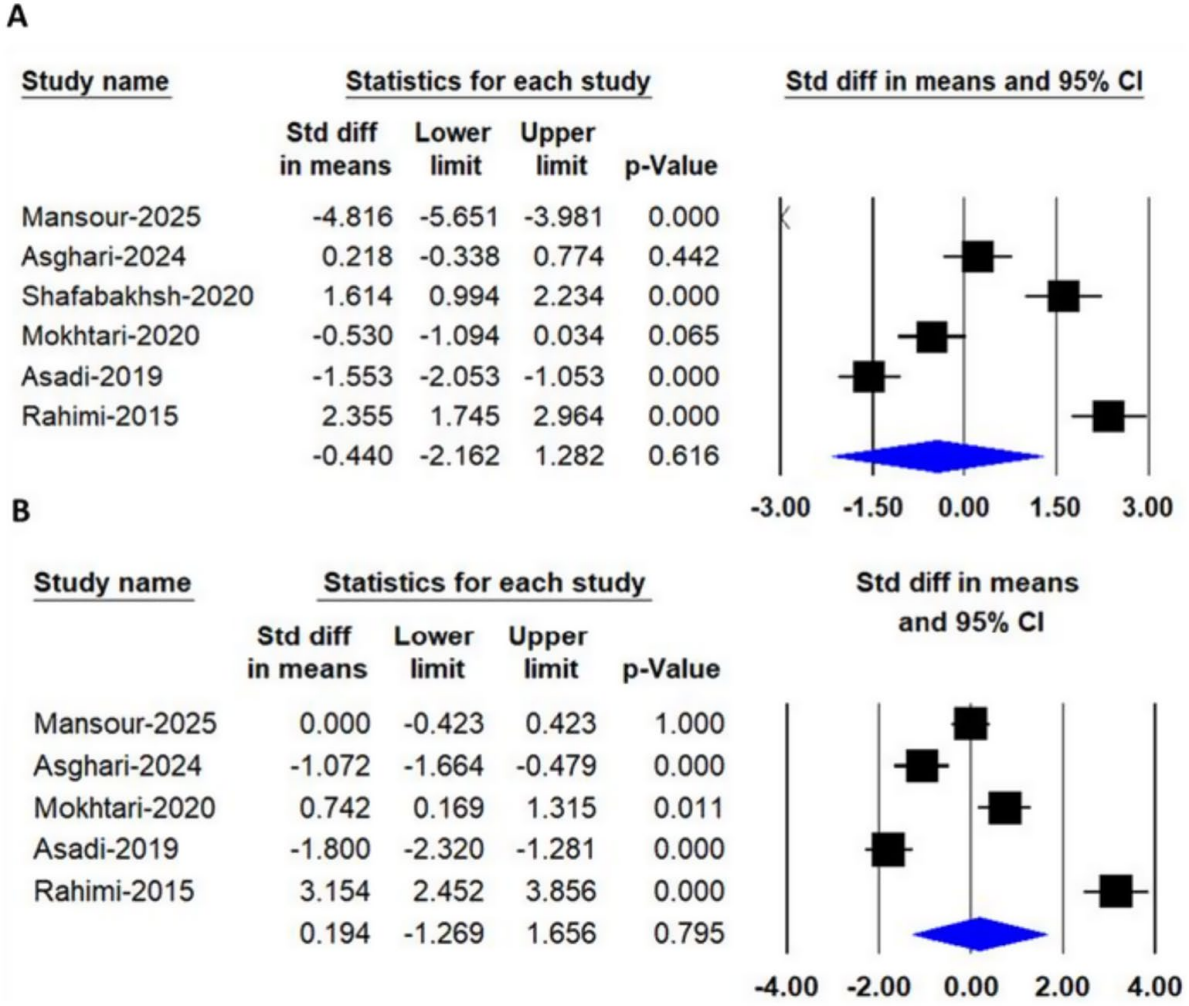
Forest plots illustrating the pooled effects of nanocurcumin supplementation on glycemic control parameters, including (A) fasting blood sugar (FBS) and (B) glycated haemoglobin (HbA1c).

A random‐effects meta‐analysis including 6 trials with a total of 389 participants (192 in the intervention group and 197 in the control group) found no significant difference in FBS levels after nanocurcumin supplementation [SMD: −0.44, 95% CI: (−2.16, 1.28), *p* = 0.61] (Figure 4).

#### 3.3.6 HbA1c

A random‐effects meta‐analysis involving 5 trials with a total of 336 participants (166 in the nanocurcumin group and 170 in the control group) showed no statistically significant difference in HbA1c levels following nanocurcumin supplementation [SMD: 0.19, 95% CI: (−1.26, 1.65), *p* = 0.79] (Figure 4).

#### 3.3.7 C‐Reactive Protein

**FIGURE 5:**
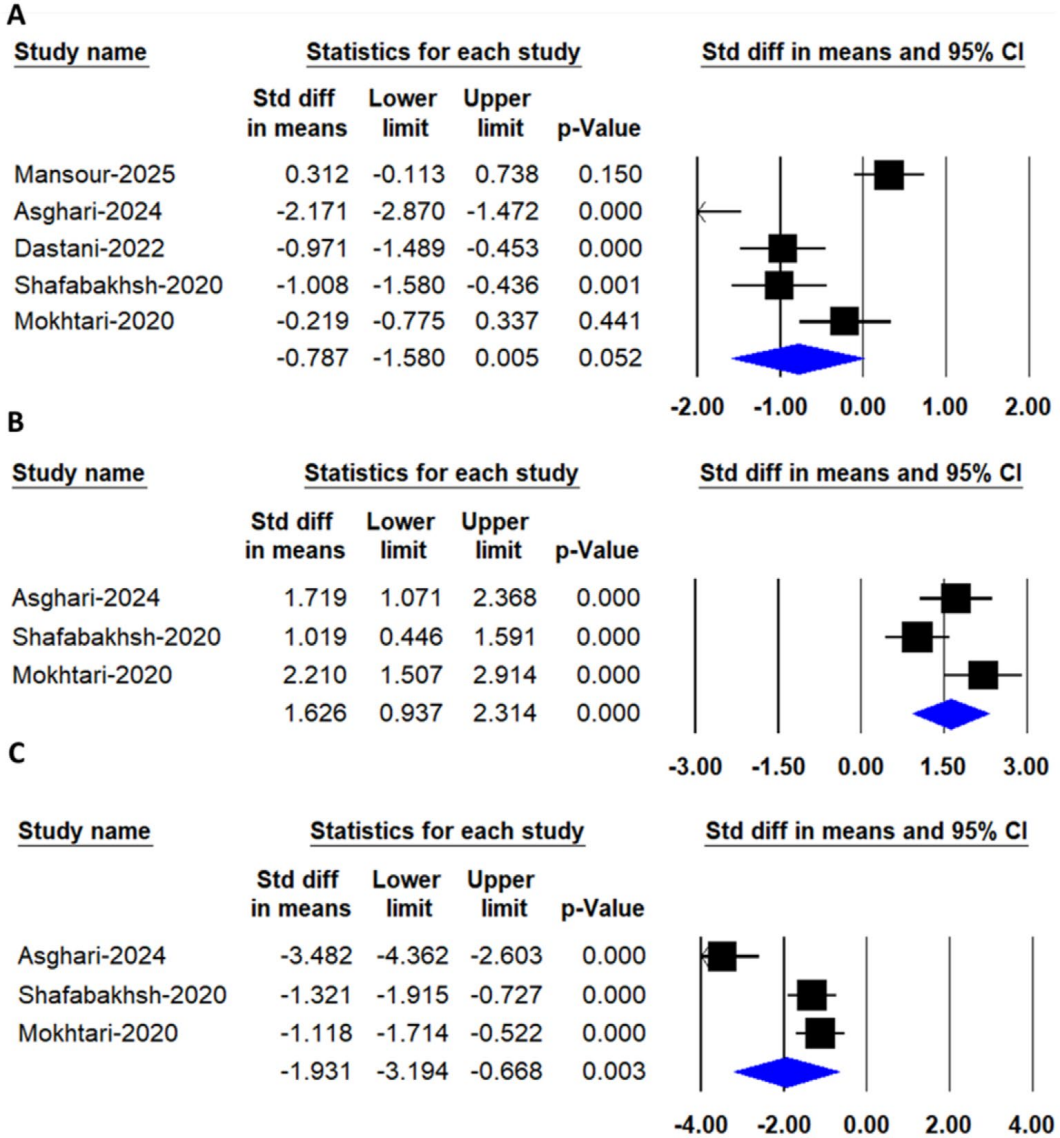
Forest plots depicting the pooled effects of nanocurcumin supplementation on biomarkers of oxidative stress and inflammation, including (A) C‐reactive protein (CRP), (B) total antioxidant capacity (TAC) and (C) malondialdehyde (MDA) levels.

A random‐effects meta‐analysis that included 5 trials with a total of 303 participants (149 cases and 154 controls) found no significant difference in C‐reactive protein (CRP) levels after consuming nanocurcumin [SMD: −0.78, 95% CI: (−1.58, 0.05), *p* = 0.052] (Figure 5).

#### 3.3.8 Total Antioxidant Capacity

A random‐effects meta‐analysis that included 3 trials with a total of 153 participants (76 cases and 77 controls) found a significant difference in total antioxidant capacity (TAC) levels after consuming nanocurcumin [SMD: 1.60, 95% CI: (0.93, 2.31), *p* = 0.00] (Figure 5).

#### 3.3.9 Malondialdehyde

A random‐effects meta‐analysis that included 3 trials with a total of 153 participants (76 cases and 77 controls) found a significant difference in Malondialdehyde (MDA) levels after consuming nanocurcumin [SMD: −1.93, 95% CI: (−3.19, −0.66), *p* = 0.03] (Figure 5).

## 4 Discussion

This systematic review and meta‐analysis of seven randomized controlled trials provides a nuanced evaluation of the effects of nanocurcumin supplementation on a spectrum of clinical parameters in patients with T2DM. The principal finding of our study is that nanocurcumin, despite its enhanced bioavailability, did not confer statistically significant benefits on conventional glycemic indices (FBS and HbA1c), lipid profiles (total cholesterol, LDL‐C, HDL‐C and triglycerides) or the inflammatory marker CRP. However, it demonstrated preliminary evidence of antioxidative effects, significantly increasing TAC and reducing MDA levels.

The findings of the present meta‐analysis should be interpreted in the context of existing evidence on curcumin and its derivatives. Previous umbrella meta‐analyses have reported that curcumin supplementation may reduce inflammatory biomarkers such as C‐reactive protein, although with substantial heterogeneity across studies [29]. Similarly, higher level evidence indicates that curcumin may improve oxidative stress markers, including reductions in malondialdehyde and modulation of antioxidant systems, although not all parameters, such as total antioxidant capacity, consistently reach statistical significance [30]. In contrast, evidence regarding lipid profile modulation remains inconsistent, with umbrella meta‐analyses suggesting only modest and variable effects depending on baseline metabolic status, dosage and study design [31]. Therefore, the lack of significant effects on glycemic control, lipid parameters and CRP observed in the present study is broadly consistent with the variability reported in prior literature. In contrast, the observed improvements in oxidative stress markers may align with the proposed antioxidative properties of curcumin, although these findings should be interpreted cautiously given the limited number of studies and high heterogeneity.

These findings indicate a differential effect, suggesting that the primary therapeutic role of nanocurcumin in T2DM may reside in its capacity to ameliorate oxidative stress, a key pathophysiological driver of diabetic complications, rather than in directly modulating glucose or lipid metabolism. These results contribute to the ongoing debate regarding the clinical utility of curcumin‐based interventions and provide a more nuanced understanding of the pharmacological profile of nanocurcumin.

Given the large variation in study designs, intervention durations, baseline metabolic control and nanocurcumin formulations, the high statistical heterogeneity observed across the included trials was expected. The variability in direction and magnitude of effects across studies further suggests that clinical and methodological diversity contributed substantially to heterogeneity. Nevertheless, despite this variability, the pooled estimates provide a summary of the average effect across diverse study conditions, which is consistent with the use of a random‐effects model.

**FIGURE 6:**
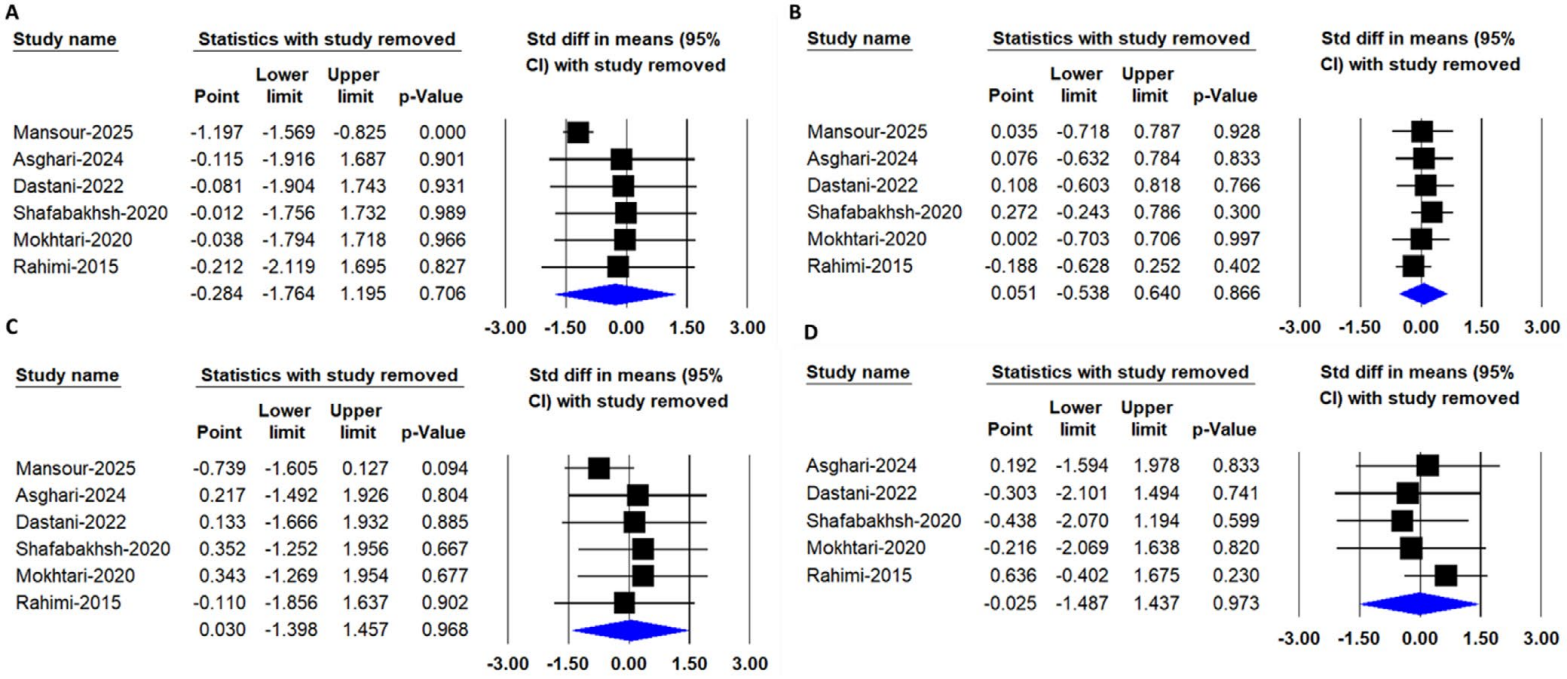
Leave‐one‐out sensitivity analysis of the pooled effects of nanocurcumin supplementation on lipid profile parameters, including (A) total cholesterol, (B) triglycerides, (C) low‐density lipoprotein cholesterol (LDL‐C) and (D) high‐density lipoprotein cholesterol (HDL‐C) levels.

**FIGURE 7:**
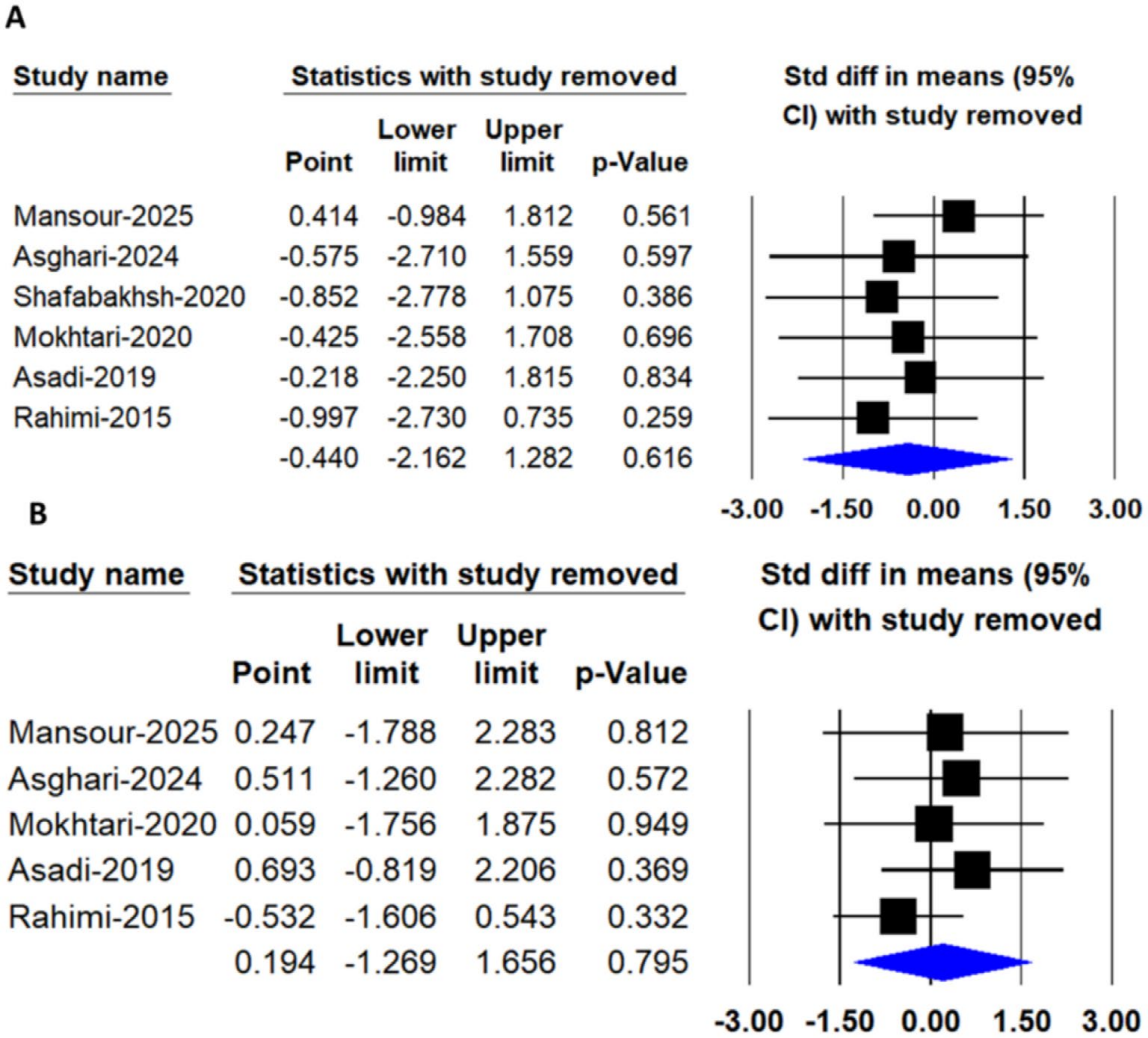
Leave‐one‐out sensitivity analysis of the pooled effects of nanocurcumin supplementation on glycemic control parameters, including (A) FBS and (B) HbA1C Levels.

**FIGURE 8:**
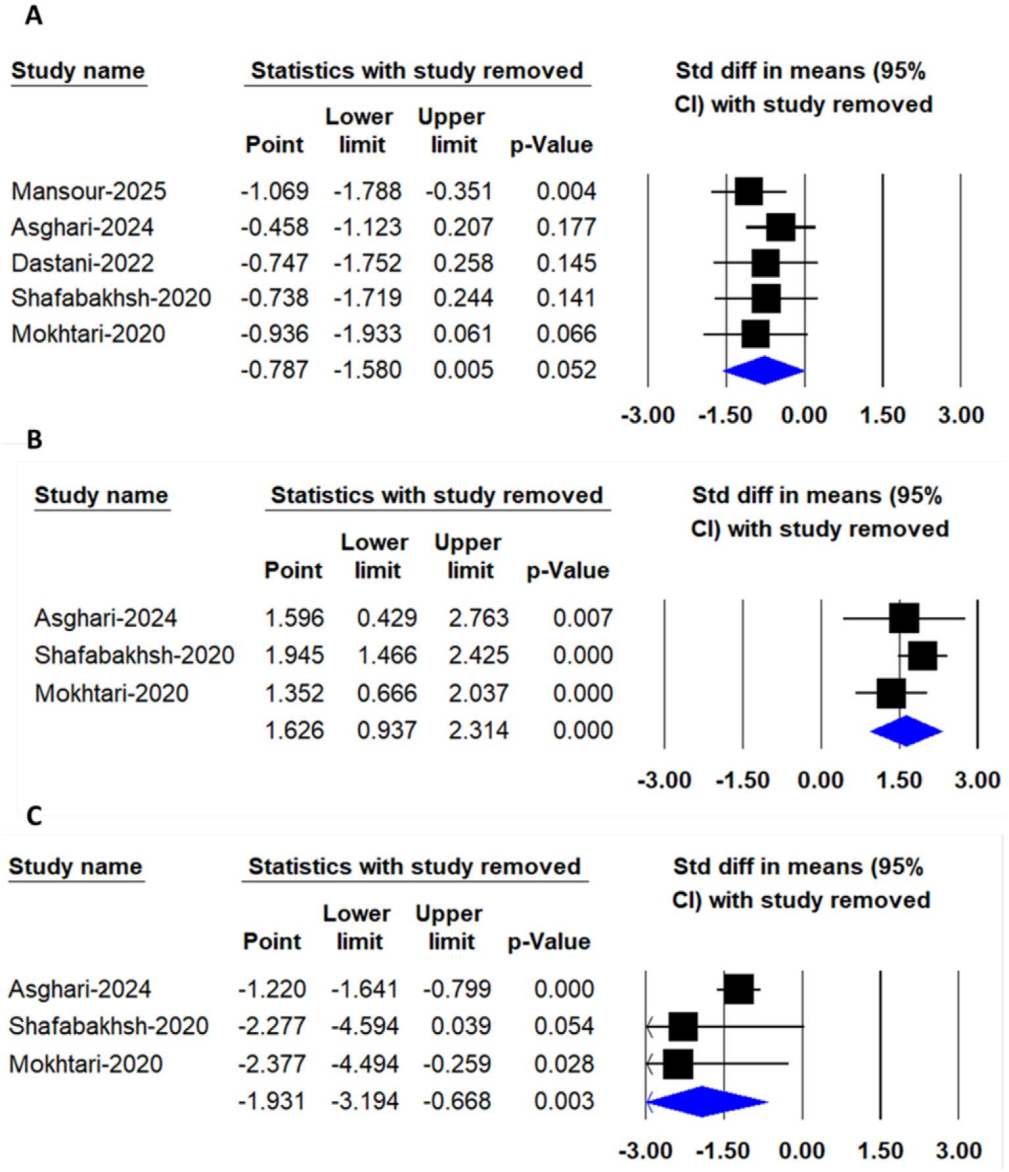
Leave‐one‐out sensitivity analysis of the pooled effects of nanocurcumin supplementation on biomarkers of inflammation and oxidative stress, including (A) C‐reactive protein (CRP), (B) total antioxidant capacity (TAC) and (C) malondialdehyde (MDA) levels.

Leave‐one‐out sensitivity analysis (Figures 6, 7, 8) revealed that the pooled effects for total cholesterol and CRP became statistically significant only after exclusion of the study by Mansour et al. [27], whereas omission of other individual studies did not materially alter the overall non‐significant findings. This observation indicates that the Mansour et al. trial exerted a disproportionate influence on these two outcomes. This influence may be explained by several distinguishing characteristics of the study, including its longer intervention duration (16 weeks compared to 8–12 weeks in other trials), inclusion of relatively older participants, and potential differences in baseline clinical status. These factors may have enhanced responsiveness to the intervention or altered the weight of the study in the pooled analysis, thereby contributing to the observed shift in statistical significance upon its exclusion.

Importantly, the Mansour et al. trial differed from other included studies in several key aspects, including a longer intervention duration (16 weeks) and an older study population. These factors may have influenced responsiveness to the intervention and contributed to the observed shift in pooled estimates upon exclusion. Therefore, this sensitivity finding highlights the impact of clinical heterogeneity, particularly differences in treatment duration and participant characteristics on the overall results. Taken together, the apparent statistical significance observed after excluding this study should be interpreted with caution and does not negate the primary pooled findings, but rather underscores the need for more homogeneous and adequately powered trials to clarify these effects.

The absence of significant glycemic improvement stands in contrast to some earlier meta‐analyses on conventional curcumin, which reported modest reductions in FBS and HbA1c [32, 33]. The lack of significant changes in glycemic indices may be attributed to the relatively short duration of intervention, insufficient dosage or variability in baseline metabolic status of participants. The included RCTs in our analysis predominantly enrolled older individuals with established T2DM who were likely on stable, optimized glucose‐lowering regimens [26, 27, 28]. Under such conditions, the marginal added benefit of an adjunctive nutraceutical may be difficult to detect, a phenomenon observed with other supplemental antioxidants [34]. Furthermore, the relatively short duration of most included trials (8–12 weeks) is a critical limitation, as it may be insufficient to detect meaningful changes in HbA1c. This is supported by real‐world evidence demonstrating that the timing of therapeutic intervention is a crucial determinant of glycemic control [35, 36].

The null effect on lipid parameters aligns with a growing body of evidence indicating that the lipid‐lowering properties of curcuminoids are inconsistent and highly dependent on baseline dyslipidemia severity and dosage [37, 38]. A recent umbrella review concluded that while curcumin may benefit certain lipid fractions, the overall effects are often modest and not universally observed across all patient cohorts [39]. Our findings corroborate this, suggesting that in medicated diabetic patients with relatively controlled baseline lipids, nanocurcumin supplementation, at the doses and durations studied, offers little additional lipid‐modifying advantage.

Despite the lack of metabolic improvements, nanocurcumin demonstrated significant beneficial effects on oxidative stress, reflected by the marked increase in TAC and reduction in MDA. These findings are partially consistent with a recent meta‐analysis showing that curcumin supplementation improves multiple oxidative stress and inflammatory biomarkers, although not all parameters, such as total antioxidant capacity, consistently reach statistical significance [30]. These findings reinforce the biological plausibility of nanocurcumin as a potent antioxidant, consistent with previous mechanistic and clinical studies demonstrating that nanoformulations significantly enhance bioavailability of curcumin, cellular uptake, and systemic exposure [18, 19, 40]. Improved oxidative parameters may influence long‐term outcomes even in the absence of immediate glycemic or lipid improvements, given that oxidative stress is a central driver of β‐cell dysfunction, endothelial injury and microvascular complications in T2DM [8, 41]. The observed improvements are therefore clinically relevant and may explain why some included trials, such as those by Asadi et al. and Mansour et al. [26, 27], reported reductions in neuropathy severity despite no improvements in glycemic indices. These organ‐specific benefits further underscore the potential therapeutic value of nanocurcumin in mitigating diabetes‐related complications through antioxidant pathways rather than by directly modulating glucose or lipid metabolism.

An important consideration is the dose–response relationship. All included trials administered 80 mg/day of nanocurcumin, which is substantially lower than doses typically used for conventional curcumin (500–1000 mg/day). Although nanoformulations improve bioavailability, the extent to which 80 mg of nanocurcumin is pharmacologically equivalent to higher doses of conventional curcumin remains unclear. It is plausible that this dosage is sufficient to exert antioxidant effects but insufficient to induce measurable metabolic changes.

Several factors may explain the absence of significant effects on glycemic and lipid outcomes. First, most included RCTs were of relatively short duration (8–12 weeks), which may be insufficient for detecting meaningful changes in HbA1c or stable lipid biomarkers. Second, although all formulations were categorized as nanocurcumin, differences in particle size, encapsulation materials and curcumin content likely contributed to between‐study heterogeneity. Third, the dosage used in most trials (80 mg/day) was lower than doses commonly associated with metabolic improvements in conventional curcumin trials (500–1000 mg/day) [31, 42]. It is possible that this lower dose, while sufficient for antioxidant effects, is sub‐therapeutic for eliciting glycemic or lipid responses in a well‐medicated cohort. These considerations highlight the need for more standardized and longer duration RCTs using optimized nanocurcumin formulations.

### 4.1 Limitations

This study has several limitations that should be acknowledged. First, the number of included randomized controlled trials (RCTs) was limited, particularly for oxidative stress markers (*n* = 3 for TAC and MDA), which may compromise the robustness of the pooled estimates. Second, substantial heterogeneity was observed across most outcomes (Table 2). Although a random‐effects model was applied, this heterogeneity likely reflects underlying clinical and methodological diversity, including differences in patient populations, concomitant treatments and outcome assessment methods.

**TABLE 2:** Assessment of heterogeneity for meta‐analysed outcomes.

Outcome	*p*‐value for heterogeneity	*I* ^2^ statistic (%)
Fasting blood sugar	< 0.001	96.92%
Haemoglobin A1C	< 0.001	95.01%
Total cholesterol	< 0.001	97.86%
Triglycerides	< 0.001	92.66%
LDL‐C	< 0.001	97.87%
HDL‐C	< 0.001	94.65%
Total antioxidant capacity	< 0.001	70.55%
Malondialdehyde	< 0.001	90.28%
C‐reactive protein	< 0.001	89.02%

A key contributor to this variability is the inconsistency in nanocurcumin formulations across studies. Important physicochemical characteristics, such as particle size, encapsulation systems, and composition, were not uniformly reported across studies (Table 3). These factors are known to significantly influence bioavailability and biological activity. Due to incomplete and uneven reporting, it was not feasible to conduct reliable subgroup or sensitivity analyses based on formulation type without introducing bias or substantially reducing statistical power. Additionally, although subgroup analysis and meta‐regression were pre‐specified, the limited number of studies and their uneven distribution across potential subgroups (e.g., identical dosing in most trials or sparse data for certain outcomes) rendered such analyses underpowered and potentially unreliable. Finally, the relatively short duration of most interventions (8–12 weeks) limits the ability to draw conclusions regarding the long‐term efficacy and safety of nanocurcumin. Future trials should adopt standardized reporting of formulation characteristics and employ longer follow‐up periods to enable more robust and clinically meaningful comparisons.

**TABLE 3:** Characteristics of nanocurcumin formulations used in the included randomized controlled trials.

	Formulation type	Particle size	Delivery system	Composition/details
Rahimi et al. [21]	Nanocurcumin (nanomicelle, SinaCurcumin)	~10 nm	Oral soft gel (nanomicellar system)	Curcumin encapsulated in nano‐micelles (≈100% encapsulation); formulated with GRAS pharmaceutical excipients; improved bioavailability vs. free curcumin
Asadi et al. [26]	Nanocurcumin capsules	Not reported	Oral capsule (nano‐formulation)	Curcumin mixture: 72% curcumin, 25% demethoxycurcumin, 3% bisdemethoxycurcumin; placebo contained polysorbate 80‐based formulation
Mokhtari et al. [25]	Nanocurcumin tablets	Not reported	Oral tablet (nano‐formulation)	80 mg/day nanocurcumin; manufacturer reported (Sina Pharmaceutical Co., Iran); detailed composition and encapsulation characteristics not reported
Sharafbakhsh et al. [24]	Nanocurcumin (nanomicelle, SinaCurcumin)	~10 nm	Oral soft gel (nanomicellar system)	Nanocurcumin (SinaCurcumin); curcuminoids encapsulated in nanomicelles with high encapsulation efficiency; improved bioavailability compared to free curcumin
Dastani et al. [28]	Nanocurcumin (nanomicellar, SinaCurcumin)	~10 nm	Oral soft gel (nanomicellar system)	80 mg/day nanocurcumin (SinaCurcumin, Exir Nano Sina); curcuminoids encapsulated in nanomicelles (~100% encapsulation); enhanced solubility and bioavailability
Asghari et al. [23]	Nanocurcumin (Sinacurcumin 80) and Omega‐3 (High EPAquatic)	Not reported	Oral capsules	Curcumin: 80 mg/day; Omega‐3: 1000 mg capsule containing 500 mg EPA and 200 mg DHA; placebos were made from oral paraffin
Mansour et al. [27]	Nanocurcumin (SinaCurcumin)	Not reported	Oral capsule	40 mg taken twice daily (80 mg total); placebo consisted of the solubilizing oil used in the nanomicelle formulation

## 5 Conclusion

In conclusion, this meta‐analysis indicates that nanocurcumin supplementation does not significantly improve glycemic control, lipid profile, or systemic inflammation in patients with type 2 diabetes mellitus within short‐term intervention periods. Although significant improvements were observed in oxidative stress markers, these findings should be interpreted with caution due to the limited number of studies, substantial heterogeneity and short duration of most trials.

Importantly, the findings of this study apply to nanocurcumin as defined by author‐reported terminology rather than a standardized or uniformly characterized formulation, given the inconsistency and incompleteness of physicochemical reporting across studies. Therefore, the observed effects may reflect a heterogeneous group of nanoformulations with potentially different bioavailability and biological activity.

Future research should prioritize well‐designed, large‐scale randomized controlled trials using standardized and fully characterized nanocurcumin formulations, with detailed reporting of physicochemical properties, to enable more precise evaluation of its clinical efficacy and to determine its impact on long‐term diabetes‐related outcomes.

## Author Contributions

**Sahar Ghoflchi:** conceptualization, writing – original draft, investigation. **Mohammad Jalili‐Nik:** investigation, writing – review and editing. **Seyed Isaac Hashemy:** investigation, writing – review and editing. **Sercan Karav:** investigation, writing – review and editing. **Hossein Hosseini:** conceptualization, writing – original draft, supervision, investigation. **Amirhossein Sahebkar:** conceptualization, writing – review and editing, supervision, investigation.

## Funding

The authors have nothing to report.

## Ethics Statement

The authors have nothing to report.

## Consent

The authors have nothing to report.

## Conflicts of Interest

The authors declare no conflicts of interest.

## Supporting information


**Figure S1:** The results of publication bias with funnel plot for (A) Cholesterol, (B) Triglycerides, (C) HDL and (C) LDL. Visual inspection of the funnel plot revealed no evidence of publication bias.

**Figure S2:** The results of publication bias with funnel plot for (A) FBA and (B) HbA1c. Visual inspection of the funnel plot revealed no evidence of publication bias.

## Data Availability

The data supporting the findings of this study are available from the corresponding author upon reasonable request.
