# Supplementary material for: Evaluating the Effects of Nanocurcumin Supplementation in Type 2 Diabetes Mellitus: A Systematic Review and Meta‐Analysis of Randomized Controlled Trials
Source: Endocrinol Diabetes Metab. 2026 May 22;9(3):e70242. doi: 10.1002/edm2.70242 (PMC13239566; doi:10.1002/edm2.70242)
Supplement: Supplementary file 1 — Figure S1: The results of publication bias with funnel plot for (A) Cholesterol, (B) Triglycerides, (C) HDL and (C) LDL. Visual inspection of the funnel plot revealed no evidence of publication bias. [file EDM2-9-e70242-s001.docx]

a


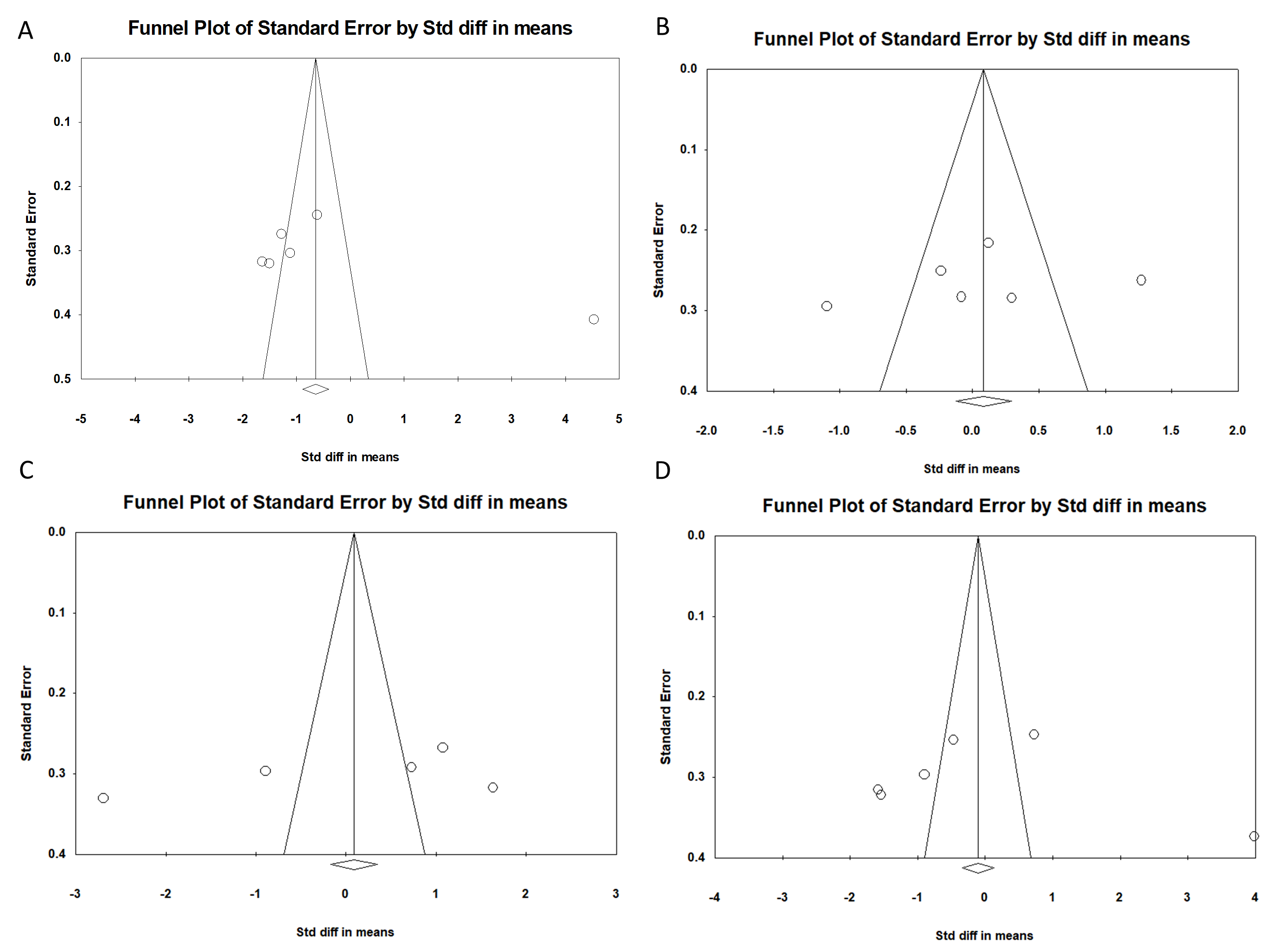


**Supplementary Fig. 1.** The results of publication bias with funnel plot for (A) Cholesterol, (B) Triglycerides, (C) HDL and (C) LDL. Visual inspection of the funnel plot revealed no evidence of publication bias.
